# Optimization of the Contrast Concentration for Low-Tube-Voltage Chest CT: A Randomized Controlled Trial with Low-Concentration Contrast Media

**DOI:** 10.3390/diagnostics16010082

**Published:** 2025-12-25

**Authors:** Jiho Park, Bo Mi Gil, Yun-Hyeon Kim, Gong Yong Jin, Yeon Joo Jeong, Jin Mo Goo, Suyon Chang, Kyongmin Sarah Beck, Jung Im Jung

**Affiliations:** 1 Department of Radiology, Seoul St. Mary’s Hospital, College of Medicine, The Catholic University of Korea, 222, Banpo-daero, Seoul 06591, Republic of Korea; hoyinside@gmail.com (J.P.); ohyes723@gmail.com (S.C.); sallahbar@gmail.com (K.S.B.); 2 Department of Radiology, Bucheon St. Mary’s Hospital, College of Medicine, The Catholic University of Korea, 327 Sosa-ro, Bucheon 14627, Republic of Korea; xhxhh@catholic.ac.kr; 3 Department of Radiology, Chonnam National University Hospital, Chonnam National University Medical School, 42 Jebong-ro, Gwangju 61469, Republic of Korea; yhkim001@jnu.ac.kr; 4 Department of Radiology, Research Institute of Clinical Medicine of Jeonbuk National University, Biomedical Research Institute of Jeonbuk National University Hospital, 20 Geongi-ro, Jeonju 54907, Republic of Korea; gyjin@jbnu.ac.kr; 5 Department of Radiology, Research Institute for Convergence of Biomedical Science and Technology, Pusan National University Yangsan Hospital, Pusan National University School of Medicine, 20 Geumo-ro, Yangsan 50612, Republic of Korea; lunar9052@hanmail.net; 6 Department of Radiology, Seoul National University College of Medicine, 101 Daehak-ro, Seoul 03080, Republic of Korea; jmgoo@snu.ac.kr

**Keywords:** tube voltage, contrast media, thorax, computed tomography

## Abstract

**Objective**: To evaluate the effect of low-concentration contrast media (LCCMs) on 100 kVp conventional chest CT by comparing the proportions of acceptable-quality scans obtained using various CM concentrations with those obtained using the standard protocol. **Materials and Methods**: This prospective, multicenter, randomized controlled trial enrolled adult patients with BMI < 30 who underwent enhanced chest CT between December 2023 and September 2024. Participants were randomly assigned to four groups: one control group (120 kVp, 320 mgI/mL) and three test groups using 100 kVp and standard CM (320 mgI/mL) or LCCMs (270 or 240 mgI/mL). Non-inferiority was tested based on the proportion of acceptable-quality scans independently assessed by two readers. Adverse reactions, including injection site pain and localized and systemic heat sensations, were recorded. **Results**: A total of 371 participants (mean age: 66.0, IQR: 58–73 years) were randomized. 100 kVp chest CT with LCCM groups showed non-inferior image quality to the standard protocol (270 mgI/mL: reader 1—98.33% confidence interval [CI]: −4.95, 9.37 and reader 2–98.33% CI: −7.11, 7.21; 240 mgI/mL: reader 1–98.33% CI: −6.44, 8.71 and reader 2–98.33% CI: −11.47, 5.11; all *p* < 0.001). Reader 2 reported a lower proportion of acceptable scans in the 320 mgI/mL low-voltage group (*p* = 0.11), whereas Reader 1 did not find significant inferiority (*p* = 0.003). Injection site pain, localized heat, and systemic heat were less frequent in LCCM groups, while systemic heat was only statistically significant (*p* = 0.003). **Conclusions**: Low-tube-voltage (100 kVp) chest CECT using LCCMs yields non-inferior image quality compared with the standard protocol while using conventional concentration contrast media under 100 kVp resulted in decreased proportion of diagnostically acceptable scans.

## 1. Introduction

Reducing the radiation dose while maintaining diagnostic accuracy is a global trend in radiology. Advances in iterative reconstruction algorithms and deep learning–based techniques have facilitated the investigation and adoption of low-voltage CT protocols [1,2]. Owing to the inherently low density and high contrast of the lung parenchyma, low-dose chest CT has rapidly emerged as the primary modality for lung assessment. Consequently, low-tube-voltage techniques, initially applied to non-enhanced chest CT, have been widely adopted for contrast-enhanced chest CT protocols. In the case of contrast-enhanced CTs, reduced tube voltage necessitates the optimization of contrast media (CM) administration. The increased photoelectric absorption of iodine at lower voltages makes scans more susceptible to soft tissue over-enhancement and contrast-related artifacts such as beam hardening and blooming [3]. Therefore, to mitigate these effects, some studies suggested reducing the iodine delivery rate by 20% when lowering the tube voltage by the same margin [4,5].

A reduction in iodine delivery can be achieved by lowering the CM concentration or injection rate. Although such adjustments may be impractical in multiphase CT protocols due to their specific requirements, chest contrast-enhanced CT (CECT) offers greater flexibility. A longer injection time is guaranteed to ensure simultaneous enhancement of the systemic veins, pulmonary arteries, and systemic arteries, with minimal dilution effects owing to target tissues’ proximity to the heart [6]. Moreover, the prevention of contrast-related artifacts is particularly important for chest CECT due to the inclusion of major vessels prone to contrast-related distortions. Despite these considerations, research on the optimization of low-voltage chest CECT protocols remains limited, and CM concentration recommendations have remained largely unchanged for more than a decade [7,8]. Recently, Beck et al. suggested that the use of low-concentration CM (LCCM) in low-voltage imaging may improve image quality by reducing beam-hardening artifacts [9]. LCCMs may provide additional benefits to patients by reducing radiation exposure and the risk of adverse events such as hypersensitivity reactions, and injection site complications [10,11].

In this context, we hypothesized that using LCCM in low-voltage conventional chest CECT may improve the image quality by minimizing unnecessary over-enhancement and contrast-related artifacts, as well as reducing the adverse events including hypersensitivity reactions. Therefore, this study aimed to identify the optimal CM concentration for 100 kVp chest CECT by comparing the proportion of diagnostically acceptable scans of 100 kVp chest CECTs obtained using varying CM concentrations against the standard protocol.

## 2. Materials and Methods

This prospective, multicenter, randomized controlled trial was approved by the institutional review board at each of six participating institutes and registered with ClinicalTrials.gov (NCT05967117). This study complied with ethical guidelines and written informed consent was obtained from all participants.

### 2.1. Study Design and Population

The image quality of chest CECTs obtained using standard protocol (120 kVp and 320 mgI/mL) and low tube voltage (100 kVp) with varying CM concentrations (240, 270, and 320 mgI/mL) were evaluated. The primary endpoint was to compare the proportion of acceptable scans acquired using the standard protocol—conventional tube voltage (120 kVp) and concentration of iodine CM (320 mgI/mL)—with those acquired using varying CM concentrations with low tube voltage.

Adult patients scheduled for chest CECT alone were recruited from six university hospitals between 9 December 2023, and 30 September 2024. Exclusion criteria included: (1) patients with a body mass index > 30; (2) aged ≤ 18 years; (3) contraindications to CECT; (4) heart failure; (5) pregnancy; (6) inability to provide informed consent; (7) anatomical variant(s) influencing image analysis; (8) history of hypersensitivity to iohexol or other iodine medications; and (9) underlying clinically significant thyroid disease(s) [12].

Participants were randomly assigned by an independent research assistant at the primary investigation center to four groups using stratified randomization based on the enrolled hospital: Group 1 (control: 120 kVp and iohexol [Iobrix, Taejoon Pharmaceutical Co., Ltd., Seoul, Republic of Korea], 320 mgI/mL), and groups 2, 3, and 4 (100 kVp and Iobrix 320, 270, and 240 mgI/mL, respectively). The evaluators were blinded to clinical and group information.

### 2.2. CT Image Acquisition

All chest CECTs were acquired using a single multidetector CT scanner (Siemens Force, Siemens Healthineers, Erlangen, Germany) (Supplementary Text S1). A total of 100 mL of CM was injected via the peripheral vein of the upper extremity at a rate of 2 mL/s with 12–20 mL of normal saline flushes. A fixed delay of 55 s was applied to ensure simultaneous enhancement of artery and vein, and minimize performer-related variation for simplified multicenter consistency. The tube current was determined by automatic exposure control using pre-scan tomography (CARE Dose4D). Scans were obtained from the thyroid to the kidney midpole. All scans were reconstructed using the iterative reconstruction algorithm (ADMIRE, level 2) with soft (Br40) and sharp kernels (Br59) for the mediastinal and lung series, respectively.

### 2.3. Image Analysis

De-identified images were uploaded to a cloud server (Bestimage, IRM, Seoul, Republic of Korea) for analysis. Each image was analyzed on a PACS workstation using dedicated software (Aquarius^®^, ver.4.4.13.P4, Terarecon, Durham, NC, USA). Assessment criteria were adopted from a previous study [9] that compared the image quality of the conventional-concentration CM versus LCCM in low-voltage chest CT. All assessments were performed independently by two radiologists (Reader 1 with 3 years; Reader 2 with 9 years of experience in thoracic imaging). To assess intra-reader variability, a repeat assessment was performed by one radiologist (Reader 1) after a 1-week washout period.

For qualitative assessment, four measures were subjectively analyzed by each radiologist mainly focusing on the body structures that can be seen in the CECT mediastinal setting images (i.e., the mediastinum, the heart, great vessels, lower neck and some of the scanned abdominal organs) and the degree of enhancement of the vessels: anatomical depiction, image noise, contrast-related artifacts, and overall diagnostic acceptability. Each parameter was evaluated on 3-, 3-, 3-, and 5-point scales according to radiologists’ decision on the image quality of mediastinal setting images. Lower scores reflected obscured anatomy, severe noise, prominent artifacts and non-diagnostic or poor quality, whereas higher scores indicated clear anatomic detail, minimal noise, artifacts, and excellent diagnostic quality. Vascular structures and thoracic soft tissues were meticulously evaluated for the adequacy of enhancement, including sufficient enhancement for clear delineation and separation of anatomical structures, as well as over-enhancement causing contrast-related artifacts or potential masking of intravascular or adjacent pathologies. Diagnostically acceptable scans were defined as those receiving a score of 3 or higher in overall diagnostic acceptability, indicating image quality that was rated as the same as or better than the standard by the radiologist. The detailed definitions of qualitative assessment scales are provided in Table S1.

Quantitative measurements were performed on the reconstructed 3.0 mm axial mediastinal images. Circular or elliptical regions-of-interest of 1.0 cm^2^ were drawn to measure the mean and standard deviation of Hounsfield units (HU) (Supplemental Text S2). The ascending aorta, descending aorta, pulmonary trunk, and erector spinae were the target tissues, with the axillary fat as the reference tissue. The signal-to-noise ratio (SNR), contrast-to-noise ratio (CNR), and figure of merit (FOM) were calculated as follows: SNR = mean attenuation/standard deviation of the tissue; CNR = (mean attenuation of the target tissue—mean attenuation of the reference tissue)/standard deviation; and FOM = CNR^2^/effective dose.

### 2.4. Patient Safety

All participants were surveyed for injection site pain and sensations of systemic or local heat after scan. Other adverse reactions potentially related to CECT were reported according to the Common Terminology Criteria for Adverse Events (CTCAE) version 5.0.

### 2.5. Statistical Analyses

The proportion of acceptable quality scans was the primary outcome. Based on previous similar studies on different CT protocols, we assumed that 90% of control scans would have acceptable quality and 10% would be of poor quality [13,14]. Non-inferiority testing of two independent proportions with a 15% non-inferiority margin was conducted. To account for multiple comparisons across three one-tailed hypotheses (group 1 vs. groups 2, 3, and 4), a standard margin of 2.5%, one-tailed type-one error was further divided into 3, making *p*-value of <0.008 to be considered statistically significant (Supplementary Text S3). Using the Non-Inferiority Tests for Two Independent Proportions in PASS 2013 with 90% statistical power, 84 participants per group (336 total) was calculated. With an additional 10% for potential dropout, the final intended sample size was 370 participants. One patient was additionally enrolled erroneously during enrollment.

Categorical variables were analyzed using the chi-square or Fisher’s exact test. Continuous variables, including baseline characteristics and secondary outcomes, were analyzed using the *t*-test or Wilcoxon rank-sum test. A *p*-value of <0.05 was considered statistically significant for secondary outcomes. To validate the assessment results, we conducted inter- and intra-reader agreement analyses using Gwet’s agreement coefficient for qualitative measures and the intra-class correlation coefficient for quantitative measures (Supplemental Text S4) [15].

All analyses were performed on a modified intention-to-treat (ITT) basis whenever feasible; otherwise, a per-protocol (PP) approach was used. SAS version 9.4 (SAS Institute Inc., Cary, NC, USA) and R version 4.3.3 were used for statistical analysis.

## 3. Results

### 3.1. Patient Population

**Figure 1:**
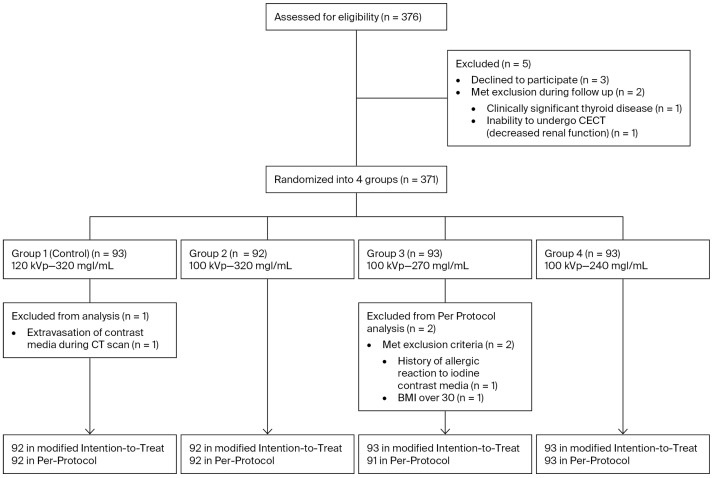
Summary of patient enrollment for the study. A total of 371 patients were enrolled due to the erroneous inclusion of one additional patient at one participating hospital. Two patients failed to be screened during enrollment and were subsequently excluded in per-protocol analysis. BMI, body mass index.

Between 9 December 2023, and 30 September 2024, a total of 376 patients provided informed consent for eligibility screening. Five participants were excluded due to withdrawal (*n* = 3) or failure to meet the inclusion criteria (*n* = 2). Ultimately, 371 patients were randomized. One patient in group 1 who underwent CT experienced contrast extravasation (*n* = 1) and was excluded. The final population for modified ITT analysis comprised 370 patients (99%). Two patients in the final population of group 3 were excluded from the PP analysis due to a history of allergic reaction to the iodine-based CM (*n* = 1) and a body mass index > 30 (*n* = 1), resulting in a final PP cohort of 368 patients (99%). Patient enrollment is illustrated in Figure 1. The indications for chest CECT were as follows: suspected malignancy or follow-up after/during treatment for a malignancy (45%), non-malignant tumors (19%), infection (9%), chronic lung disease (8%), and others (19%). The demographics and clinical status were generally well balanced among the four groups (Table 1).

### 3.2. Image Analysis

**Figure 2:**
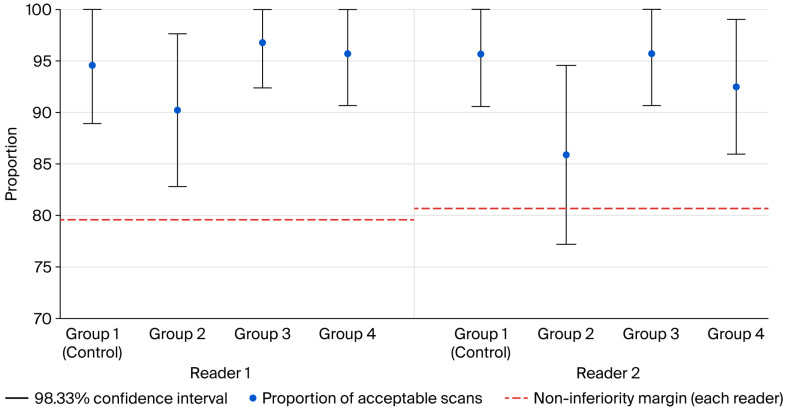
Proportion of acceptable scans and non-inferiority margins in the modified intention-to-treat population. The non-inferiority margin is defined as a 15% reduction in acceptable scans in the test group. A 98.33% confidence interval is applied to each group to test three distinct one-tailed non-inferiority testing hypotheses with a *p*-value threshold of 0.0083. A lower confidence interval crossing the non-inferiority margin indicates potential inferiority of the result.

**Figure 3:**
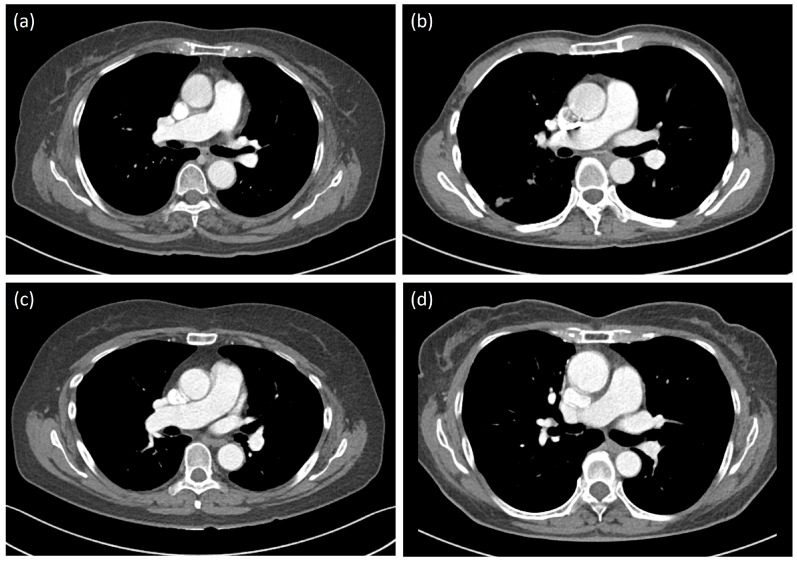
Representative images of each group in the contrast-enhanced axial 3 mm mediastinal setting at the level of the pulmonary hilum (window width, 400; window level, 40). Group 1: 120 kVp, 320 mgI/mL (**a**); group 2: 100 kVp, 320 mgI/mL (**b**); group 3: 100 kVp, 270 mgI/mL (**c**); and group 4: 100 kVp, 240 mgI/mL (**d**).

In terms of the proportion of acceptable scans (primary outcome), 100 kVp scans with LCCMs (groups 3 and 4) showed non-inferior results compared with the control group in both modified ITT and PP analyses by both readers (*p* < 0.001). Both groups also reported slightly higher proportions of acceptable scans. Although group 2 showed a lower proportion of acceptable scans than the control group, only the assessment by the second reader failed to meet the non-inferiority threshold (*p* = 0.11), indicating group 2 was inferior to standard group. These results were consistent with those of the modified ITT and PP analyses, ensuring the robustness of the results. The primary outcome results are summarized in Table 2 and Figure 2, with representative images shown in Figure 3.

Regarding qualitative metrics, there was no difference in the level of anatomical depiction between the readers (*p* = 0.55 and 0.13, respectively). Reader 1 reported significant differences in image noise among the groups (*p* = 0.04), with the lowest image noise reported in group 1 (8.7%, negligible image noise); however, reader 2 reported no such differences (*p* = 0.88). Both readers reported differences in contrast-related artifacts (*p* = 0.01). They both reported the highest ratio of scans with severe contrast-related artifacts in group 2. Group 2 also recorded the highest proportions of scans rated as suboptimal or limited in overall diagnostic acceptability by both readers (9.8% and 14.1%, respectively), with the result from reader 2 being statistically significant (*p* = 0.04). Detailed analysis results are summarized in Table S2.

**Figure 4:**
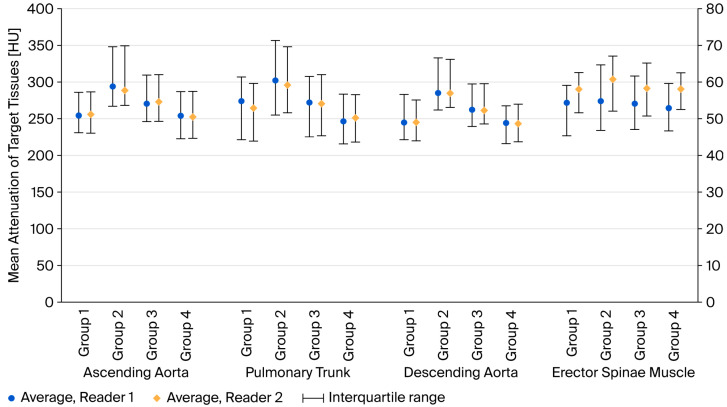
Measured mean attenuation of target tissues in each group by two readers. The average of mean attenuation is indicated by points (circle and diamond) with interquartile ranges. Three vessels (ascending aorta, pulmonary trunk, and descending aorta) show increased attenuation in groups 2 and 3, whereas similar attenuation is observed in groups 4 and 1 (control). The erector spinae show a similar average attenuation in each group. The results from two readers are consistent.

In the quantitative analysis, both readers showed consistent results. Most values, including the mean attenuation, standard deviation, SNR, CNR, and FOM, of the selected tissues differed among the groups. Measurements by both readers showed that the mean attenuation of the ascending aorta, descending aorta, and pulmonary trunk was higher in group 2 than in group 1. Group 3 exhibited similar or slightly higher attenuation values than the control group, whereas group 4 showed no difference (*p* value of post hoc analysis for group 4 versus group 1: reader 1 > 0.999, 0.746, 0.106 and reader 2 > 0.999, 0.945, 0.414 for ascending aorta, descending aorta and pulmonary trunk, respectively). The erector spinae, which represents the soft tissues of interest to clinicians and radiologists, showed no difference in the mean attenuation or FOM among the groups. Detailed analysis results are summarized in Table S3. Figure 4 depicts the mean attenuation of target tissues in each group.

Inter- and intra-reader analysis for qualitative analysis using Gwet’s agreement coefficient indicated substantial to almost perfect agreement between the readers (range: 0.63–0.98), suggesting consistent grading of CT scans regardless of the reader and time (correlation coefficient: >0.80, almost perfect; 0.60–0.79, substantial; 0.40–0.59, moderate; 0.20–0.39, fair; and <0.20, none to slight agreement). For the quantitative analysis, the intraclass correlation coefficient ranged from poor to excellent reliability. The mean attenuation of the three vessels showed excellent reliability (>0.90), whereas the erector spinae and axillary fat showed poor to good reliability (range: 0.28–0.81) (intraclass correlation coefficient: <0.50, poor; 0.50–0.75, moderate; 0.75–0.90, good; and >0.90, excellent reliability) [14]. Factors such as differences in the muscle fat content, partial volume effects from adjacent vessels, and anatomical heterogeneity may have influenced measurement consistency. The results of agreement and reliability analyses are presented in Tables S4–S7.

### 3.3. Radiation

All three test groups that underwent low-voltage imaging experienced a lower radiation dose in terms of the CT dose index, dose–length product value, and effective dose (*p* < 0.001). The scan length differed among the groups and was slightly shorter in group 1 (*p* = 0.04). Further details are presented in Table S8.

### 3.4. Patient Safety

Patients who underwent CT with LCCMs (groups 3 and 4) reported a lower incidence of systemic heat (*p* = 0.003; 53.8%, 50.5%, 39.8% for 320, 270, 240 mgI/mL, respectively). Exploratory post hoc analysis showed that, compared with the conventional concentration group (320 mgI/mL), the 240-mgI/mL group experienced less systemic heat. Furthermore, group 4 reported the lowest incidence of injection site heat, and patients who received LCCMs reported fewer instances of injection site pain, although these differences were not statistically significant (*p* = 0.34 and 0.41, respectively, 18.5%, 18.3%, 11.8% for local heat sensation; 10.3%, 6.5%, 6.5% for injection site pain in 320, 270, 240 mgI/mL respectively).

The study was not powered to detect statistically significant differences in less frequent adverse events. Only four patients experienced adverse events other than injection-site pain or heat, all of which were categorized as mild or moderate according to CTCAE v5.0. A full summary of adverse events is provided in Table 3.

## 4. Discussion

This study aimed to compare the proportion of acceptable-quality scans obtained using 100 kVp chest CECT and varying concentrations of iodine CM with that obtained using the standard protocol to identify the optimal CM concentration for 100 kVp chest CT. Primary outcome analysis by both readers suggested (modified ITT) that 100 kVp chest CECT with LCCMs (270 and 240 mgI/mL) yielded results comparable to the standard protocol (120 kVp and 320 mgI/mL). The mean attenuation and FOM of the erector spinae in the low-concentration groups did not significantly differ from those of the control group, indicating comparable soft tissue depiction despite reduced iodine delivery. Adequate aortic enhancement, conventionally defined as 150–200 HU in chest CT, was achieved in both LCCM groups, with the mean attenuation exceeding 200 HU [16].

Conversely, readers reported a higher proportion of poor-quality scans in group 2 (320 mgI/mL), although only reader 2’s evaluation showed statistical inferiority. The difference in the statistical significance between two readers might have stemmed from difference in their length of experience (3 yrs vs. 9 yrs). However, both readers’ decreased proportion of acceptable scans in group 2 may be attributed to the increased number of contrast-related artifacts [3,4]. At 100 kVp, the increased photoelectric absorption of iodine substantially elevated vascular attenuation in Group 2 (mean ≈ 300 HU), which produced marked over-enhancement and a higher prevalence of beam-hardening and streak artifacts. By contrast, both LCCM groups (240 and 270 mgI/mL) delivered 16–25% less iodine, resulting in more physiologic intravascular enhancement (mean ≈ 250–270 HU), effectively mitigating over-enhancement and reducing artifact severity. This finding justifies the need for LCCMs in low-tube-voltage protocol. And it aligns with our hypothesis that pairing low tube voltage with a standard-concentration CM leads to excessive intravascular enhancement, increased artifacts, and ultimately a reduction in diagnostic acceptability.

Beck et al. [9] previously reported that low-voltage Chest CT can maintain image quality when iodine concentration is reduced. However, this study was consecutive, observational study with heterogeneous scan methods (five different scanners, mixed iterative reconstruction usage, and varying tube voltage of 120, 100, and 80 kVp). In addition, the tested LCCM was limited to 240 mgI/mL. To validate and extend Dr. Beck’s findings, we designed a prospective multicenter, randomized trial with two available LCCMs (270 mgI/mL and 240 mgI/mL) under tightly controlled acquisition conditions to provide stronger evidence. We believe that our study confirms that the use of LCCMs in low-tube voltage protocol objectively preserves image quality and provides insight that using standard-concentration CM at low kVp may lead to over-enhancement and increased artifacts.

For the two different LCCMs used in our study, both demonstrated non-inferior image quality, but potential trade-off were observed. Group 3 (270 mgI/mL) showed a slightly higher proportion of acceptable scans (e.g., 96.77% for Reader 1), while group 4 (240 mgI/mL) showed the lowest incidence of systemic heat and its vascular attenuation values were most comparable to those of the control group. These findings suggest that both concentrations are clinically acceptable; however, selecting the optimal LCCM dose requires balancing image quality against patient comfort and iodine minimization. Further tailored evaluation is warranted to determine the most appropriate concentration for specific clinical needs.

Lowering the iodine concentration in CT offers additional benefits, including improved patient safety and cost-effectiveness. The 100 kVp groups experienced lower radiation doses than control group. Furthermore, these groups demonstrated a lower incidence of adverse events. The incidence of systemic heat was significantly lower (*p* = 0.003), with the lowest frequency observed in the 240-mgI/mL group (*p* = 0.01 in post hoc analysis vs. 320-mgI/mL group). Although the incidences of local heat sensation and injection site pain were lower (18.5%, 18.3%, 11.8% for local heat sensation; 10.3%, 6.5%, 6.5% for injection site pain for 320, 270, 240 mgI/mL, respectively), the differences were not statistically significant (*p* = 0.34 and 0.41, respectively). Previous studies have similarly highlighted the potential of LCCMs to reduce the incidence of adverse events [10,11]. The lack of statistical significance may stem from the relatively small sample size and low incidence of adverse events in this study. Further research is needed to investigate the effects of LCCMs on adverse events. Our findings suggest that patients undergoing repetitive low-tube-voltage and LCCM imaging, particularly for oncologic purposes, will benefit from reduced cumulative radiation exposure and a lower risk of adverse events.

This study has several limitations. First, the non-inferiority assessment was based on scan acceptability, not diagnostic outcomes, due to practical constraints such as the prolonged follow-up required to establish definitive diagnoses. Nonetheless, a robust assessment framework encompassing various imaging parameters—image noise, anatomical depiction, enhancement, and artifacts—was used to support scan quality assessments. Second, all chest CECT scans were performed on a single CT scanner from a single vendor using highly standardized conventional enhanced chest CT protocols with 55 sec fixed delays. Therefore, the generalizability of our findings to other scanners, imaging protocols, and dedicated vascular indications—such as CT pulmonary angiography requires further investigation. Third, we evaluated the scan quality using only mediastinal images, excluding the lung parenchyma. However, as contrast is typically unnecessary for assessing pulmonary structures owing to their intrinsic high contrast and chest CECT is largely reserved for the evaluation of mediastinal, vascular, or other soft tissue structures, our study focused on evaluating the image quality of the most clinically relevant regions. Fourth, our study included adult patients who underwent chest CT alone, whereas in clinical practice, multi-region CT scans are common. Therefore, the applicability of LCCM to other anatomical regions and simultaneous multi-part CT acquisition protocols warrant further investigation. Furthermore, the potential benefits of LCCMs in pediatric populations—where low-tube-voltage CT is advantageous—should also be explored.

Moreover, all examinations were performed using a single fixed injection rate of 2 mL/s. Because injection rate interacts with contrast concentration and total iodine delivery, our findings may not be generalizable to protocols that use higher or variable injection rates. Future studies should explore the combined effects of concentration, volume, and injection rate. Also, we excluded subjects with a high BMI (BMI > 30) to minimize the influence of body weight on image quality. Because low-kVp CT with reduced iodine concentration is generally recommended for low-to-average-sized patients [17,18], and our cohort excluded individuals with BMI > 30 to minimize body-size–related confounding, the present findings therefore are most applicable to this population.

For tube voltage, 100 kVp was selected as a representative example of low tube voltage due to resource constraints, institutional practices, and its widespread use in thoracic imaging. While automated tube selection offers a broader range, our results are consistent with previous reports suggesting a 20% reduction in iodine delivery can be achieved with a 20% reduction in tube voltage. Our study demonstrated that a 16–25% reduction in iodine delivery (from 320 mgI/mL to 240–270 mgI/mL) with 17% voltage reduction (from 120 kVp to 100 kVp) maintained non-inferior image quality [4]. Nevertheless, further investigation is necessary to refine CM dosing across different voltage settings. Finally, although adverse events were systematically recorded, the study was not powered to detect statistically significant differences in less frequent adverse events, and non-significant findings should be interpreted with caution. Larger studies are needed to more definitively assess the safety profile of LCCMs, particularly regarding rare adverse reactions.

## 5. Conclusions

In conclusion, 100 kVp chest CECT using LCCMs (270 or 240 mgI/mL) produced a non-inferior proportion of acceptable-quality scans compared with the standard protocol while preserving soft tissue and vascular enhancement. By contrast, 100 kVp imaging with a conventional CM concentration (320 mgI/mL) reduced the proportion of acceptable scans. The findings in this study may serve as a guide for optimizing image quality in low-tube-voltage chest CT through tailored selection of CM concentration. This approach also has potential to reduce costs and enhance patient safety in routine thoracic imaging.

## Figures and Tables

**Table 1:** Demographic and clinical characteristics of the study population.

	All (N = 370)	Group 1 (Control), 320 mgI/mL and 120 kVp (n = 92)	Group 2, 320 mgI/mL and 100 kVp (n = 92)	Group 3, 270 mgI/mL and 100 kVp (n = 93)	Group 4, 240 mgI/mL and 100 kVp (n = 93)	*p*-Value
**Demographics**						
Age	66.0 (58.0–73.0)	66.0 (57.0–73.0)	66.5 (56.0–72.0)	66.0 (59.0–73.0)	66.0 (57.0–75.0)	0.94
Sex						0.27
male	238 (64.3)	54 (58.7)	57 (62.0)	60 (64.5)	67 (72.0)	
female	132 (35.7)	38 (41.3)	35 (38.0)	33 (35.5)	26 (28.0)	
Body mass index	23.4 ± 3.3	23.5 ± 2.8	23.6 ± 3.1	23.2 ± 3.7	23.2 ± 3.5	0.83
Smoking						0.14
current smoker	68 (18.4)	10 (10.9)	17 (18.5)	22 (23.7)	19 (20.4)	
ex-smoker	145 (39.2)	35 (38.0)	32 (34.8)	36 (38.7)	42 (45.2)	
never smoker	157 (42.4)	47 (51.1)	43 (46.7)	35 (37.6)	32 (34.4)	
**Vital Sign**						
Systolic blood pressure	128.0 (120.0–137.0)	129.5 (123.0–142.0)	128.0 (120.5–135.5)	128.0 (121.0–138.0)	125.0 (118.0–135.0)	0.18
Diastolic blood pressure	78.0 (70.0–84.0)	78.5 (72.0–83.5)	77.5 (70.0–83.0)	78.0 (70.0–85.0)	76.0 (70.0–84.0)	0.95
Pulse rate	79.0 (72.0–88.0)	78.0 (72.0–88.0)	80.5 (75.5–89.0)	78.0 (72.0–86.0)	80.0 (72.0–88.0)	0.31
Respiratory rate	18.0 (16.0–20.0)	18.0 (16.0–20.0)	18.0 (16.0–20.0)	18.0 (16.0–20.0)	18.0 (16.0–20.0)	0.53
**Comorbidities**						
Hypertension	135 (36.5)	27 (29.3)	35 (38.0)	35 (37.6)	38 (40.9)	0.40
Diabetes mellitus	78 (21.1)	17 (18.5)	18 (19.6)	24 (25.8)	19 (20.4)	0.62
Stroke	8 (2.2)	2 (2.2)	1 (1.1)	3 (3.2)	2 (2.2)	0.96
Coronary artery disease	21 (5.7)	4 (4.3)	4 (4.3)	3 (3.2)	10 (10.8)	0.11
Thyroid disease	25 (6.8)	7 (7.6)	7 (7.6)	5 (5.4)	6 (6.5)	0.92
Asthma	21 (5.7)	8 (8.7)	1 (1.1)	5 (5.4)	7 (7.5)	0.12
Chronic kidney disease	4 (1.1)	0 (0.0)	2 (2.2)	1 (1.1)	1 (1.1)	0.62
Allergies	17 (4.6)	4 (4.3)	4 (4.3)	7 (7.5)	2 (2.2)	0.41

Note: Data are presented as n (%), median (interquartile range), or mean ± standard deviation. The *p*-values were calculated using the chi-square test, Fisher’s exact test, analysis of variance, or Kruskal–Wallis test.

**Table 2:** Proportion and comparison of acceptable scans in each group.

	Number of Acceptable Scans	Proportion of Acceptable Scans (98.3% CI)	Risk Difference (98.3% CI)	*p*-Value
Modified ITT				
Reader 1				
Group 1	87/92	94.57 (88.91, 100.00)		
Group 2	83/92	90.22 (82.80, 97.63)	−4.35 (−13.67, 4.98)	0.003
Group 3	90/93	96.77 (92.39, 100.00)	2.21 (−4.95, 9.37)	<0.001
Group 4	89/93	95.70 (90.66, 100.00)	1.13 (−6.44, 8.71)	<0.001
Reader 2				
Group 1	88/92	95.65 (90.56, 100.00)		
Group 2	79/92	85.87 (77.18, 94.56)	−9.78 (−19.85, 0.29)	0.11
Group 3	89/93	95.70 (90.66, 100.00)	0.05 (−7.11, 7.21)	<0.001
Group 4	86/93	92.47 (85.93, 99.02)	−3.18 (−11.47, 5.11)	<0.001
Per protocol				
Reader 1				
Group 1	87/92	94.57 (88.91, 100.00)		
Group 2	83/92	90.22 (82.80, 97.63)	−4.35 (−13.67, 4.98)	0.003
Group 3	88/91	96.70 (92.22, 100.00)	2.14 (−5.08, 9.35)	<0.001
Group 4	89/93	95.70 (90.66, 100.00)	1.13 (−6.44, 8.71)	<0.001
Reader 2				
Group 1	88/92	95.65 (90.56, 100.00)		
Group 2	79/92	85.87 (77.18, 94.56)	−9.78 (−19.85, 0.29)	0.11
Group 3	88/91	96.70 (92.22, 100.00)	1.05 (−5.73, 7.83)	<0.001
Group 4	86/93	92.47 (85.93, 99.02)	−3.18 (−11.47, 5.11)	<0.001

Note: The number of acceptable scans is presented as the total number in each group. The proportion of acceptable scans and risk differences are presented with a 98.3% confidence interval (CI). The *p*-value was calculated using the non-inferiority test of proportions in comparison with the control group (group 1). A *p*-value of <0.008 was considered statistically significant. A per-protocol analysis was conducted to verify the robustness of the modified intention-to-treat analysis. ITT, modified intention-to-treat; PP, per-protocol; group 1, 120 kVp and 320 mgI/mL; group 2, 100 kVp and 320 mgI/mL; group 3, 100 kVp and 270 mgI/mL; group 4, 100 kVp and 240 mgI/mL.

**Table 3:** Summary of adverse events in the test population.

	320 mgI/mL (n = 184)	270 mgI/mL (n = 93)	240 mgI/mL (n = 93)	*p*-Value	Group-Wise Comparison (*p*-Value) Using Bonferroni Correction		
					320 vs. 270	320 vs. 240	320 vs. LCCM
Injection site pain				0.41	0.86	0.86	0.54
No pain	165 (89.7)	87 (93.5)	87 (93.5)				
Pain	19 (10.3)	6 (6.5)	6 (6.5)				
Systemic heat				0.003	0.07	0.01	<0.001
None	85 (46.2)	46 (49.5)	56 (60.2)				
Mild heat	59 (32.1)	39 (41.9)	32 (34.4)				
Moderate heat	39 (21.2)	8 (8.6)	5 (5.4)				
Severe heat	1 (0.5)	0 (0.0)	0 (0.0)				
Injection site heat	34 (18.5)	17 (18.3)	11 (11.8)	0.34	>0.999	0.468	>0.999
Adverse effects	2 (100.0)	2 (100.0)	-	N/A	N/A	N/A	N/A
CTCAE term				>0.999	>0.999	N/A	>0.999
Nausea	1 (50.0)	1 (50.0)	-				
Urinary urgency	0 (0.0)	1 (50.0)	-				
Urticaria	1 (50.0)	0 (0.0)	-				
CTCAE grade				>0.999	>0.999	N/A	>0.999
1	1 (50.0)	2 (100.0)	-				
2	1 (50.0)	0 (0.0)	-				

Note: Data are presented as n (%). The *p*-values were calculated using the chi-square test or Fisher’s exact test. CTCAE, Common Terminology Criteria for Adverse Events; LCCM, low-concentration contrast media (270 mgI/mL and 240 mgI/mL).

## Data Availability

The data presented in this study are available on request from the corresponding author. The data are not publicly available due to privacy and ethical restrictions, as the dataset contains patient-related information.

## Supplementary Materials

The following supporting information can be downloaded at: https://www.mdpi.com/article/10.3390/diagnostics16010082/s1, Supplementary Text S1: Details of the CT acquisition protocol; Supplementary Text S2: Tissue location selection for quantitative analysis; Supplementary Text S3: The *p*-value threshold for non-inferiority testing; Supplementary Text S4: Inter- and intra-reader agreement analysis; Table S1: Qualitative analysis scales for anatomical depiction, noise, contrast-related artifacts, and overall diagnostic acceptability; Table S2: Qualitative analysis results; Table S3: Quantitative analysis results; Table S4: Inter- and intra-reader agreement analysis of qualitative measures using Gwet’s agreement coefficient; Table S5: Reference values for the interpretation of Gwet’s agreement coefficient; Table S6: Inter- and intra-reader agreement analysis of quantitative measures using the ICC; Table S7: Reference values for the interpretation of the intra-class correlation coefficient. Table S8: Comparing Radiation Between Groups.

## Abbreviations

The following abbreviations are used in this manuscript:

LCCM	Low-concentration contrast media
CM	Contrast media
CECT	Contrast-enhanced computed tomography
HU	Hounsfield units
SNR	Signal-to-noise ratio
CNR	Contrast-to-noise ratio
FOM	Figure-of-merit
CTCAE	Common Terminology Criteria for Adverse Events
