# Supplementary material for: Optimization of the Contrast Concentration for Low-Tube-Voltage Chest CT: A Randomized Controlled Trial with Low-Concentration Contrast Media
Source: Diagnostics (Basel). 2025 Dec 25;16(1):82. doi: 10.3390/diagnostics16010082 (PMC12785640; doi:10.3390/diagnostics16010082)
Supplement: Supplementary file 1 [file diagnostics-16-00082-s001.zip › diagnostics-4022753-supplementary.pdf]

Supplemental material

Optimization of the contrast concentration for low-tube-voltage chest CT: A randomized controlled trial with low-concentration contrast media

Appendix and tables

Supplementary Text S1. Details of the CT acquisition protocol

All CTs scans analyzed in this study were obtained using a single multidetector CT system (Siemens Force) with a single breath-hold technique. As Tin filter is used for ultra-low-voltage CTs, no Tin filter was used in our protocol. The scan range extended from the thyroid to the midpole of the kidney. Automatic exposure control was performed using pre-scan tomography (CARE Dose4D) was used. All scans were reconstructed using an iterative reconstruction algorithm (ADMIRE, factor x2) at 3-mm and 1-mm slice thicknesses. A soft kernel (Br40) was used for the mediastinum, and a sharp kernel (Br59) was used for the lung setting. For administering the contrast, a total of 100 mL of contrast solution was injected at a rate of 2mL/s after a 12-mL saline flush. This was followed by a 20-mL saline flush. Contrast-enhanced chest scans were obtained 55 s acquired with a fixed 55-s delay to capture the venous phase.

Supplementary Text S2. Tissue location selection for quantitative analysis

Two readers independently measured the mean attenuation and standard deviation of the reference and target tissues by drawing 1.0-cm^2 sized circular or elliptical regions of interest in the reference and target tissues. Optimal locations were determined by each reader to best represent the characteristics of the tissues, excluding artifacts and other tissues, such as calcifications and fat. No specific guidelines, such as specific spinal levels, were provided to the readers.

Supplementary Text S3. The *p*-value threshold for non-inferiority testing

The non-inferiority test was performed to compare the proportion of acceptable scans in each group with that in the control group (120 kVp, 320 mgI/mL). As we planned to perform three independent one-tailed (non-inferiority) tests for each of the three test groups, the threshold for statistical significance was set as follows for the primary outcome:

$$0.05 \div 2 \text{ (for one} - \text{tailed hypothesis)} \div 3 \text{ (three independent hypothesis testing)} = 0.0083$$

Supplementary Text S4. Inter- and intra-reader agreement analysis

Gwet's agreement coefficient was used in this study because of the well-established problem with Cohen's kappa, known as the paradox of Cohen's kappa [1]. This phenomenon occurs in the case of high-prevalence data and was expected to occur in this study, as all scans were performed in tertiary referral centers with highly standardized protocols. For further justification, we present the results in parallel with the percentage agreement in this study.

Reference

[1] Zec S, Soriani N, Comoretto R, Baldi I. High agreement and high prevalence: The paradox of Cohen's kappa. Open Nurs J 2017;11:211-218. https://doi.org/10.2174/1874434601711010211

Table S1. Qualitative analysis scales for anatomical depiction, noise, contrast-related artifacts, and overall diagnostic acceptability

Scales	Anatomical depiction	Image noise	Contrast-related artifacts	Overall diagnostic acceptability	Quality of the scan
1	Obscured anatomic detail, and enhancement is not sufficient for diagnosis	Severe image noise affecting the visualization of normal structures	Severe contrast-related artifacts affecting the visualization of normal structures	Non-diagnostic image quality	Poor quality
2	Anatomic detail is clear, and most images display a high degree of vascular enhancement	Minor image noise not affecting the visualization of normal structures	Minor contrast-related artifacts affecting the visualization of normal structures	Suboptimal or limited image quality	
3	Distinct anatomic detail, and high degree of vascular opacification	Negligible image noised in any structure	Negligible contrast-related artifacts in any structure	Standard image quality	Acceptable quality
4				Better than the standard image quality	
5				Excellent image quality	

Note: The quality of the scan is defined according to the overall diagnostic acceptability. Poor quality is defined as non-diagnostic, suboptimal, or limited image quality. Acceptable quality is defined as standard, better than standard, and excellent image quality.

Table S2. Qualitative analysis results

	Group 1 (n=92)	Group 2 (n=92)	Group 3 (n=93)	Group 4 (n=93)	*p*-value	Bonferroni correction for the multiple comparisons		
						p-value[a] group 2 vs. group 1	*p*-value[b] group 3 vs. group 1	*p*-value[c] group 4 vs. group 1
**Radiologist 1 (initial)**								
Anatomical depiction					0.551	>0.999	>0.999	>0.999
Moderate	2 (2.2)	5 (5.4)	2 (2.2)	2 (2.2)				
Excellent	90 (97.8)	87 (94.6)	91 (97.8)	91 (97.8)				
Noise					0.037	0.299	0.055	0.173
Severe	-	-	-	-				
Minor	84 (91.3)	90 (97.8)	92 (98.9)	91 (97.8)				
Negligible	8 (8.7)	2 (2.2)	1 (1.1)	2 (2.2)				
Artifact					0.011	>0.999	0.015	>0.999
Severe	3 (3.3)	6 (6.5)	3 (3.2)	2 (2.2)				
Minor	68 (73.9)	66 (71.7)	49 (52.7)	63 (67.7)				
Negligible	21 (22.8)	20 (21.7)	41 (44.1)	28 (30.1)				
Overall diagnostic acceptability					0.104	>0.999	0.356	>0.999
Non-diagnostic	-	-	-	-				
Suboptimal or limited	5 (5.4)	9 (9.8)	3 (3.2)	4 (4.3)				
Standard	60 (65.2)	61 (66.3)	49 (52.7)	60 (64.5)				
Better than standard	25 (27.2)	22 (23.9)	40 (43.0)	28 (30.1)				
Excellent	2 (2.2)	0 (0.0)	1 (1.1)	1 (1.1)				
Quality of the scan					0.233	0.798	>0.999	>0.999
Poor quality	5 (5.4)	9 (9.8)	3 (3.2)	4 (4.3)				
Acceptable quality	87 (94.6)	83 (90.2)	90 (96.8)	89 (95.7)				

**Radiologist 2**								
Anatomical depiction					0.133	0.178	>0.99	0.720
Moderate	4 (4.3)	11 (12.0)	4 (4.3)	8 (8.6)				
Excellent	88 (95.7)	81 (88.0)	89 (95.7)	85 (91.4)				
Noise					0.881	>0.99	>0.99	N/A
Severe	0 (0.0)	1 (1.1)	1 (1.1)	0 (0.0)				
Minor	92 (100.0)	91 (98.9)	92 (98.9)	93 (100.0)				
negligible	-	-	-	-				
Artifact					0.012	0.168	0.244	>0.999
Severe	2 (2.2)	10 (10.9)	1 (1.1)	4 (4.3)				
Minor	79 (85.9)	71 (77.2)	70 (75.3)	72 (77.4)				
Negligible	11 (12.0)	11 (12.0)	22 (23.7)	17 (18.3)				
Overall diagnostic acceptability					0.040	0.209	0.486	>0.999
Non-diagnostic	-	-	-	-				
Suboptimal or limited	4 (4.3)	13 (14.1)	4 (4.3)	7 (7.5)				
Standard	77 (83.7)	68 (73.9)	68 (73.1)	70 (75.3)				
Better than standard	11 (12.0)	11 (12.0)	21 (22.6)	16 (17.2)				
Excellent	-	-	-	-				
Quality of the scan					0.038	0.066	>0.999	>0.999
Poor quality	4 (4.3)	13 (14.1)	4 (4.3)	7 (7.5)				
Acceptable quality	88 (95.7)	79 (85.9)	89 (95.7)	86 (92.5)				

Data are presented as n (%). The *p*-values were calculated using the chi-squared test or Fisher's exact test.

[a] Comparison between groups 1 and 2. [b] Comparison between groups 1 and 3. [c] Comparison between groups 1 and 4.

Group 1, 120 kVp and 320 mgI/mL; group 2, 100 kVp and 320 mgI/mL; group 3, 100 kVp and 270 mgI/mL; group 4, 100 kVP and 240 mgI/mL.

Table S3. Quantitative analysis results

	Group 1, 320 mgI/mL and 120 kVP	Group 2, 320 mgI/mL and 100 kVP	Group 3, 270 mgI/mL and 100 kVP	Group 4, 240 mgI/mL and 100 kVP	*p*-value	Bonferroni correction for the multiple comparisons[a]		
						p value[a]	p value[b]	p value[c]
**Radiologist 1 (first)**	n=92	n=92	n=92	n=92				
Ascending aorta								
Mean Hounsfield unit (HU)	254.5 (231.0–286.0)	294.0 (267.0–348.5)	270.5 (246.0–309.5)	254.0 (223.0–287.0)	<0.001	<0.001	0.089	>0.999
SD of HU	16.3 (14.9–18.3)	18.5 (17.1–20.3)	18.1 (16.5–19.7)	17.9 (16.6–19.1)	<0.001	<0.001	0.001	0.001
Signal-to-noise ratio	16.0 (13.8–18.3)	16.1 (14.2–18.3)	15.3 (12.8–18.0)	14.3 (12.2–15.7)	<0.001	>0.999	0.515	0.003
Contrast to noise ratio	24.3 (20.4–28.5)	23.6 (20.3–28.4)	21.4 (18.7–24.5)	20.5 (17.8–24.5)	<0.001	>0.999	0.002	<0.001
Figure of merit	377.7 (256.9–596.1)	405.7 (289.7–682.5)	353.9 (251.3–538.5)	333.8 (193.8–486.5)	0.009	0.566	>0.999	0.122
Descending aorta								
Mean HU	245.0 (221.5–283.0)	285.5 (262.0–333.0)	262.5 (239.5–297.5)	244.5 (216.0–268.0)	<0.001	<0.001	0.034	0.746
SD of HU	17.4 (15.6–18.7)	19.6 (17.8–21.7)	18.6 (16.9–20.4)	18.5 (16.6–21.0)	<0.001	<0.001	0.001	0.001
Signal-to-noise ratio	14.2 (12.8–16.3)	14.7 (13.0–17.4)	14.1 (12.3–16.2)	12.9 (11.3–14.6)	<0.001	0.856	>0.999	0.001
Contrast to noise ratio	24.1 (20.1–27.3)	22.7 (20.0–28.0)	20.7 (18.3–24.8)	19.9 (17.2–23.9)	<0.001	>0.999	0.004	<0.001
Figure of merit	363.1 (248.9–545.3)	386.0 (269.1–662.5)	346.8 (243.0–506.4)	307.4 (186.4–472.0)	0.005	0.493	>0.999	0.117
Pulmonary trunk								
Mean HU	274.0 (221.5–307.0)	302.5 (255.0–356.5)	272.0 (225.0–308.0)	246.5 (215.5–283.5)	<0.001	0.001	>0.999	0.106

SD of HU	16.8 (15.0–18.3)	18.8 (16.8–20.8)	18.1 (16.3–20.1)	17.9 (16.3–19.1)	<0.001	<0.001	0.002	0.022
Signal-to-noise ratio	16.0 (13.8–18.6)	16.3 (13.7–18.5)	15.1 (12.1–16.8)	13.7 (12.4–15.9)	<0.001	>0.999	0.130	0.001
Contrast to noise ratio	25.2 (21.3–30.4)	24.3 (19.7–27.7)	21.3 (18.3–25.4)	19.9 (16.8–25.2)	<0.001	0.719	0.001	<0.001
Figure of merit	422.9 (263.5–608.9)	402.7 (270.4–690.3)	374.9 (231.0–536.3)	304.4 (187.4–512.2)	0.002	>0.999	0.409	0.020
Erector spinae								
Mean HU	54.4 (45.4–59.1)	54.8 (46.8–64.7)	54.1 (47.1–61.6)	52.9 (46.7–59.6)	0.371	0.515	>0.999	>0.999
SD of HU	20.4 (17.8–23.0)	23.0 (19.7–26.1)	21.6 (19.5–24.2)	23.2 (20.9–25.6)	<0.001	<0.001	0.039	<0.001
Signal-to-noise ratio	2.7 (2.1–3.1)	2.5 (1.8–3.0)	2.5 (1.9–3.2)	2.2 (1.9–2.7)	0.046	0.443	0.879	0.012
Contrast to noise ratio	10.4 (8.8–12.0)	9.2 (8.0–10.6)	9.1 (7.8–10.4)	9.2 (7.8–10.4)	<0.001	0.007	0.002	0.001
Figure of merit	72.0 (52.1–92.0)	66.2 (45.5–94.4)	63.9 (45.9–86.6)	63.4 (43.7–86.4)	0.238	>0.999	0.477	0.172
Axillary fat								
Mean HU	-97.1 (-104.0 to -91.3)	-104.0 (-110.0 to -98.0)	-104.0 (-110.0 to -96.6)	-105.0 (-110.0 to -97.0)	<0.001	<0.001	<0.001	<0.001
SD of HU	14.9±3.0	17.4±3.0	17.6±3.3	17.4±3.1	<0.001	<0.001	<0.001	<0.001
Signal-to-noise ratio	-6.6 (-8.1 to -5.7)	-5.9 (-7.0 to -5.1)	-6.1 (-7.1 to -4.9)	-6.1 (-7.0 to -5.2)	0.007	0.016	0.014	0.017
**Radiologist 2**	n=92	n=92	n=92	n=92				
Ascending aorta								
Mean HU	255.5 (230.0–285.5)	288.5 (267.5–346.5)	272.5 (246.0–309.0)	252.0 (223.5–283.5)	<0.001	<0.001	0.023	>0.999
SD of HU	17.4 (15.7–19.1)	19.0 (17.0–20.8)	17.8 (16.1–19.7)	18.6 (16.9–20.2)	0.001	0.001	0.602	0.014
Signal-to-noise ratio	14.9 (12.9–17.1)	15.5 (13.2–18.4)	15.5 (12.8–18.0)	13.6 (11.9–15.9)	<0.001	0.183	0.830	0.039
Contrast to noise ratio	24.6 (21.1–28.5)	23.4 (20.3–26.8)	21.8 (19.1–24.9)	21.9 (18.4–25.3)	<0.001	0.800	0.003	0.002

Figure of merit	343.0 (266.5–614.9)	413.6 (300.5–637.8)	383.7 (262.6–516.8)	310.4 (219.4–532.6)	0.026	0.516	>0.999	0.225
Descending aorta								
Mean HU	245.5 (220.0–275.5)	285.0 (265.5–331.0)	261.5 (243.0–298.0)	243.5 (218.5–270.0)	<0.001	<0.001	0.010	0.945
SD of HU	17.4±2.4	19.7±2.6	18.8±2.2	18.5±3.0	<0.001	<0.001	<0.001	0.012
Signal-to-noise ratio	14.1 (12.5–16.6)	14.5 (13.1–17.1)	14.4 (12.3–15.8)	12.8 (11.9–15.2)	0.001	0.587	>0.999	0.017
Contrast to noise ratio	24.1 (20.6–27.9)	22.7 (19.9–26.5)	21.2 (18.5–24.8)	21.4 (18.0–25.0)	<0.001	0.971	0.003	0.001
Figure of merit	328.0 (260.0–594.6)	396.1 (288.2–585.0)	340.8 (260.4–504.2)	294.9 (207.0–509.5)	0.012	0.420	>0.999	0.160
Pulmonary trunk								
Mean HU	264.0 (219.0–298.0)	295.5 (258.0–348.0)	270.5 (227.0–310.0)	251.5 (218.0–283.0)	<0.001	0.001	>0.999	0.414
SD of HU	17.1 (15.4–19.4)	18.7 (17.2–21.0)	18.2 (16.5–20.0)	18.0 (16.7–19.6)	<0.001	<0.001	0.113	0.142
Signal-to-noise ratio	15.6 (13.1–18.0)	15.4 (13.2–18.5)	14.6 (12.3–17.9)	13.6 (11.8–15.8)	0.002	>0.999	0.714	0.006
Contrast to noise ratio	25.6 (21.1–30.0)	24.6 (19.9–27.0)	20.8 (18.4–26.2)	21.1 (18.3–25.1)	<0.001	0.462	0.001	<0.001
Figure of merit	386.8 (266.6–653.5)	404.7 (299.6–684.3)	349.6 (244.4–513.9)	303.9 (206.0–578.5)	0.013	>0.999	0.503	0.071
Erector spinae								
Mean HU	56.9±8.4	59.6±10.1	58.4±9.7	57.6±6.9	0.239	0.148	0.793	>0.999
SD of HU	19.1 (17.2–21.7)	20.5 (19.0–22.9)	20.0 (18.2–22.7)	20.2 (18.3–22.7)	0.006	0.006	0.158	0.060
Signal-to-noise ratio	3.0±0.8	2.9±0.8	3.0±0.8	2.9±0.6	0.358	0.818	>0.999	0.261
Contrast to noise ratio	10.9±2.5	9.7±2.1	9.6±2.1	9.9±2.0	<0.001	0.001	<0.001	0.008
Figure of merit	76.2 (56.1–105.3)	72.4 (53.4–93.6)	68.5 (47.2–97.0)	71.8 (48.6–104.6)	0.501	>0.999	0.490	0.758
Axillary fat								

Mean HU	-100.0 (-106.0 to -93.1)	-106.0 (-111.0 to -102.0)	-105.0 (-111.0 to -98.6)	-105.0 (-111.0 to -101.0)	<0.001	<0.001	0.001	<0.001
SD of HU	14.4 (12.8–16.6)	17.0 (15.7–19.3)	17.0 (15.1–19.6)	16.5 (14.8–19.0)	<0.001	<0.001	<0.001	<0.001
Signal-to-noise ratio	-6.9±1.7	-6.2±1.5	-6.2±1.4	-6.4±1.4	0.001	0.004	0.004	0.059

Note: Data are presented as the median (interquartile range [IQR]) or mean ± standard deviation (SD). The *p*-values were calculated using the analysis of variance or Kruskal–Wallis test.

[a] Comparison between groups 1 and 2. [b] Comparison between groups 1 and 3. [c] Comparison between groups 1 and 4.

Table S4. Inter- and intra-reader agreement analysis of qualitative measures using Gwet's agreement coefficient

Radiologist 1 (first)	Inter-rater agreement (radiologists 1 and 2)		intra-rater agreement (radiologist 1st and 2nd)	
	Percentage of agreement (95% confidence interval [CI])	Gwet's agreement coefficient 1 (95% CI)	Percentage of agreement (95% CI)	Gwet's agreement coefficient 1 (95% CI)
**Group 1, standard: 320 mgI/mL and 120 kVP**				
Anatomical depiction	0.93 (0.86, 0.98)	0.93 (0.88, 0.99)	0.98 (0.92, 1.00)	0.98 (0.95, 1.00)
Noise	0.91(0.84,0.96)	0.91 (0.85, 0.97)	0.83 (0.73, 0.90)	0.81 (0.72, 0.90)
Artifact	0.79 (0.70, 0.87)	0.77 (0.67, 0.86)	0.89 (0.81, 0.95)	0.87 (0.80, 0.95)
Overall	0.90 (0.82, 0.95)	0.89 (0.82, 0.97)	0.95 (0.88, 0.98)	0.94 (0.89, 0.99)
**Group 2, 320 mgI/mL and 100 kVP**				
Anatomical depiction	0.85 (0.76, 0.91)	0.83 (0.75, 0.92)	0.90 (0.82, 0.95)	0.90 (0.83, 0.96)
Noise	0.97 (0.91,0.99)	0.97 (0.93, 1.00)	0.98 (0.92, 1.00)	0.98 (0.95, 1.00)
Artifact	0.79 (0.70, 0.87)	0.76 (0.66, 0.86)	0.85 (0.76, 0.91)	0.82 (0.74, 0.91)
Overall	0.83 (0.73, 0.90)	0.78 (0.67, 0.89)	0.90 (0.82, 0.95)	0.89 (0.81, 0.96)
**Group 3, 270 mgI/mL and 100 kVP**				
Anatomical depiction	0.94 (0.86, 0.98)	0.93 (0.88, 0.99)	0.98 (0.92,1.00)	0.98 (0.95, 1.00)
Noise	0.98 (0.92,1.00)	0.98 (0.95, 1.00)	0.98 (0.92, 1.00)	0.98 (0.95,1.00)
Artifact	0.69 (0.58, 0.78)	0.63 (0.52, 0.74)	0.88 (0.80, 0.94)	0.86 (0.78, 0.94)
Overall	0.95 (0.88, 0.98)	0.94 (0.89, 0.99)	0.97 (0.91, 0.99)	0.97 (0.93, 1.00)
**Group 4, 240 mgI/mL and 100 kVP**				
Anatomical depiction	0.89 (0.81, 0.95)	0.89 (0.82, 0.96)	0.98 (0.92, 1.00)	0.98 (0.95, 1.00)
Noise	0.98 (0.92, 1.00)	0.98 (0.95,1.00)	0.97 (0.91, 0.99)	0.97 (0.93, 1.00)
Artifact	0.84 (0.75, 0.91)	0.81 (0.72, 0.90)	0.97 (0.91, 0.99)	0.96 (0.92, 1.00)
Overall	0.92 (0.85, 0.97)	0.92 (0.85, 0.98)	0.97 (0.91, 0.99)	0.97 (0.93, 1.00)

Table S5. Reference values for the interpretation of Gwet's agreement coefficient

Agreement coefficient	Strength of agreement
<0.20	None to slight agreement
0.20–0.39	Fair agreement
0.40–0.59	Moderate agreement
0.60–0.79	Substantial agreement
>0.80	Almost perfect agreement

Table S6. Inter- and intra-reader agreement analysis of quantitative measures using the ICC

	Values of target and reference tissues			Inter-rater agreement (radiologists 1 and 2)	intra-rater agreement (radiologist 1st and 2nd)
	Radiologist 1 (first)	Radiologist 2	Radiologist 1 (second)	ICC (95% CI)	ICC (95% CI)
**Group 1, standard: 320 mgI/mL and 120 kVP**	n=92	n=92	n=92		
Ascending aorta					
Mean attenuation	260.7±42.9	257.4±42.5	259.2±43.4	0.96 (0.94, 0.98)	0.98 (0.97, 0.99)
Standard deviation	16.7±2.6	17.5±2.6	17.1±2.4	0.63 (0.46, 0.75)	0.54 (0.38, 0.67)
Signal-to-noise ratio	15.9±3.3	15.0±3.0	15.4±3.0	0.77 (0.61, 0.86)	0.72 (0.60, 0.80)
Contrast-to-noise ratio	24.9±6.1	24.9±6.1	25.1±5.7	0.65 (0.52, 0.75)	0.69 (0.57, 0.79)
Figure of merit	465.4±303.0	463.2±297.3	465.5±294.0	0.81 (0.72, 0.87)	0.80 (0.72, 0.86)
Descending aorta					
Mean attenuation	251.2±42.0	250.5±41.9	251.5±41.0	0.99 (0.98, 0.99)	0.99 (0.99, 1.00)
Standard deviation	17.2±2.1	17.4±2.4	17.1±2.1	0.49 (0.32, 0.63)	0.56 (0.41, 0.69)
Signal-to-noise ratio	14.8±3.0	14.7±3.1	14.9±2.9	0.81 (0.72, 0.87)	0.84 (0.77, 0.89)
Contrast-to-noise ratio	24.3±6.0	24.4±6.0	24.6±5.5	0.66 (0.53, 0.76)	0.69 (0.57, 0.78)
Figure of merit	443.0±296.7	447.0±295.1	445.7±277.6	0.82 (0.74, 0.88)	0.80 (0.71, 0.86)
Pulmonary trunk					
Mean attenuation	268.0±59.5	264.7±60.0	268.0±59.7	0.99 (0.98, 0.99)	0.99 (0.99, 1.00)
Standard deviation	17.0±2.4	17.4±2.7	16.9±2.3	0.54 (0.39, 0.67)	0.73 (0.62, 0.81)
Signal-to-noise ratio	16.0±3.7	15.5±3.7	16.0±3.6	0.80 (0.71, 0.86)	0.89 (0.84, 0.93)
Contrast-to-noise ratio	25.5±6.9	25.5±6.9	25.8±6.4	0.75(0.64, 0.82)	0.75 (0.65, 0.83)
Figure of merit	495.4±351.4	494.2±331.8	496.7±310.7	0.84 (0.77, 0.89)	0.81 (0.72, 0.87)
Erector spinae					
Mean attenuation	51.5±18.5	56.9±8.4	51.7±10.7	0.29 (0.09, 0.46)	0.38 (0.19, 0.54)
Standard deviation	20.8±4.8	19.4±3.2	20.7±5.1	0.47 (0.28, 0.62)	0.66 (0.52, 0.76)
Signal-to-noise ratio	2.6±1.4	3.0±0.8	2.7±0.9	0.23 (0.04, 0.41)	0.37(0.18, 0.53)
Contrast-to-noise ratio	10.3±3.0	10.9±2.5	10.5±2.1	0.39(0.21, 0.55)	0.39 (0.21, 0.55)
Figure of merit	77.6±37.6	83.6±40.2	76.9±34.1	0.67 (0.54, 0.77)	0.66 (0.53, 0.76)
Axillary fat					
Mean attenuation	-95.4±17.7	-98.8±9.3	-97.4±8.6	0.28 (0.09, 0.46)	0.30(0.10, 0.47)
Standard deviation	14.9±3.0	14.9±3.1	14.6±2.3	0.39 (0.20, 0.55)	0.52(0.36, 0.66)
Signal-to-noise ratio	-6.7±1.9	-6.9±1.7	-6.9±1.5	0.46 (0.28, 0.61)	0.49 (0.32, 0.63)
**Group 2, 320 mgI/mL and 100 Kvp**	n=92	n=92	n=92		
Ascending aorta					
Mean attenuation	305.0±59.8	304.2±53.3	306.9±51.8	0.87(0.81, 0.91)	0.89 (0.84, 0.93)
Standard deviation	18.9±2.4	19.0±2.6	18.6±2.1	0.62 (0.48, 0.73)	0.44 (0.26, 0.59)

Signal-to-noise ratio	16.4±3.7	16.4±4.0	16.7±3.2	0.77 (0.67, 0.84)	0.69 (0.57, 0.78)
Contrast-to-noise ratio	24.3±5.8	24.3±6.0	24.7±6.0	0.60 (0.45, 0.72)	0.71(0.59, 0.80)
Figure of merit	533.7±352.9	540.5±382.6	560.2±393.9	0.80 (0.71, 0.86)	0.83 (0.76, 0.89)
Descending aorta					
Mean attenuation	296.3±52.1	296.9±51.1	297.2±49.7	0.97 (0.95, 0.98)	0.97 (0.95, 0.98)
Standard deviation	19.8±2.7	19.7±2.6	20.0±2.4	0.40 (0.21, 0.56)	0.39 (0.20, 0.55)
Signal-to-noise ratio	15.3±3.3	15.4±3.4	15.1±3.2	0.78 (0.68, 0.85)	0.71 (0.59, 0.80)
Contrast-to-noise ratio	23.8±5.5	23.8±5.8	24.1±5.8	0.61 (0.47, 0.73)	0.74 (0.63, 0.82)
Figure of merit	511.2±337.1	521.6±365.4	534.3±371.3	0.80 (0.71, 0.86)	0.85 (0.78, 0.90)
Pulmonary trunk					
Mean attenuation	309.0±75.6	305.7±75.3	308.4±74.1	0.99 (0.98, 0.99)	0.99(0.99, 1.00)
Standard deviation	19.0±2.9	19.4±3.4	19.0±2.4	0.50 (0.33, 0.64)	0.56(0.41, 0.69)
Signal-to-noise ratio	16.5±4.2	16.0±4.4	16.4±3.8	0.85 (0.77, 0.90)	0.84(0.77, 0.89)
Contrast-to-noise ratio	24.5±6.4	24.3±6.5	24.8±6.6	0.67 (0.54, 0.77)	0.78(0.69, 0.85)
Figure of merit	544.9±389.2	541.4±393.6	565.8±416.4	0.77 (0.67, 0.84)	0.84(0.77, 0.89)
Erector spinae					
Mean attenuation	55.3±12.5	59.6±10.1	51.8±19.6	0.51 (0.32, 0.66)	0.42 (0.24, 0.57)
Standard deviation	23.4±4.5	21.1±3.4	54.7±300.0	0.32 (0.11, 0.51)	0.00 (-0.20, 0.20)
Signal-to-noise ratio	2.5±0.9	2.9±0.8	2.3±1.2	0.42 (0.19, 0.59)	0.47 (0.30, 0.62)
Contrast-to-noise ratio	9.4±2.0	9.7±2.1	9.4±2.4	0.34(0.14, 0.50)	0.46 (0.28, 0.61)
Figure of merit	76.5±39.8	81.1±42.7	77.7±41.2	0.65 (0.51, 0.75)	0.74 (0.63, 0.82)
Axillary fat					
Mean attenuation	-103.0±9.9	-103.4±18.8	-103.4±18.8	0.37 (0.19, 0.54)	0.42 (0.24, 0.57)
Standard deviation	17.4±3.0	17.4±3.1	17.1±2.7	0.34 (0.15, 0.51)	0.61 (0.47, 0.73)
Signal-to-noise ratio	-6.2±1.5	-6.2±1.5	-6.2±1.5	0.46 (0.28, 0.60)	0.61 (0.46, 0.72)
**Group 3, 270 mgI/mL and 100 kVP**	n=92	n=92	n=92		
Ascending aorta					
Mean attenuation	273.9±44.7	274.6±44.5	273.2±45.1	0.95 (0.92, 0.97)	0.97 (0.96, 0.98)
Standard deviation	18.3±3.1	18.1±2.7	18.3±2.8	0.55 (0.39, 0.68)	0.44 (0.26, 0.59)
Signal-to-noise ratio	15.4±3.5	15.5±3.4	15.2±3.1	0.75 (0.65, 0.83)	0.76 (0.66, 0.84)
Contrast-to-noise ratio	22.2±5.2	22.2±4.3	22.4±4.2	0.52 (0.35, 0.65)	0.62 (0.48, 0.73)
Figure of merit	414.3±240.8	410.5±202.8	418.4±192.2	0.56 (0.40, 0.68)	0.66 (0.53, 0.76)
Descending aorta					
Mean attenuation	264.8±39.7	266.6±38.8	265.8±39.3	0.93 (0.90, 0.95)	0.94 (0.91, 0.96)
Standard deviation	18.6±2.6	18.8±2.2	18.5±2.3	0.50 (0.33, 0.64)	0.52 (0.36, 0.66)
Signal-to-noise ratio	14.5±3.0	14.3±2.6	14.6±2.8	0.77 (0.68, 0.84)	0.77 (0.67, 0.84)
Contrast-to-noise ratio	21.6±4.8	21.8±4.3	22.0±4.1	0.53 (0.37, 0.66)	0.60 (0.45, 0.72)
Figure of merit	391.3±214.6	395.5±204.5	402.0±185.3	0.58 (0.42, 0.70)	0.66 (0.53, 0.76)
Pulmonary trunk					
Mean attenuation	271.1±59.6	271.2±60.0	269.9±59.6	0.97 (0.95, 0.98)	0.98 (0.97, 0.99)

Standard deviation	18.4±3.1	18.3±2.6	18.0±2.6	0.56 (0.40, 0.68)	0.35 (0.16, 0.52)
Signal-to-noise ratio	15.1±4.0	15.1±4.1	15.3±4.2	0.84 (0.77, 0.89)	0.83 (0.76, 0.89)
Contrast-to-noise ratio	22.1±6.0	22.1±5.6	22.4±5.5	0.72 (0.61, 0.81)	0.76 (0.66, 0.83)
Figure of merit	406.9±230.7	407.8±219.6	416.3±213.3	0.68 (0.55, 0.78)	0.73 (0.62, 0.82)
Erector spinae					
Mean attenuation	54.0±10.4	58.4±9.7	53.3±10.9	0.58 (0.35, 0.73)	0.70 (0.57, 0.79)
Standard deviation	23.7±16.8	20.5±3.7	22.8±4.1	0.00 (-0.20, 0.20)	0.05 (-0.15, 0.25)
Signal-to-noise ratio	2.5±0.8	3.0±0.8	2.5±0.8	0.33 (0.11, 0.52)	0.48 (0.31, 0.62)
Contrast-to-noise ratio	9.3±2.2	9.6±2.1	9.4±2.1	0.58 (0.42, 0.70)	0.64 (0.50, 0.74)
Figure of merit	69.8±34.1	75.6±37.9	71.7±32.6	0.54 (0.38, 0.67)	0.62 (0.48, 0.73)
Axillary fat					
Mean attenuation	-102.3±11.0	-104.1±10.4	-102.4±10.2	0.66 (0.53, 0.76)	0.81 (0.73, 0.87)
Standard deviation	17.6±3.3	17.6±3.7	17.2±3.1	0.47 (0.30, 0.62)	0.59 (0.44, 0.71)
Signal-to-noise ratio	-6.1±1.6	-6.2±1.4	-6.2±1.4	0.60 (0.45, 0.72)	0.68 (0.55, 0.78)
**Group 4, 240 mgI/mL and 100 kVP**	n=92	n=92	n=92		
Ascending aorta					
Mean attenuation	254.6±39.6	253.7±39.4	254.0±40.3	0.97 (0.95, 0.98)	0.98 (0.97, 0.99)
Standard deviation	17.9±2.3	18.5±2.3	17.9±2.6	0.42 (0.24, 0.57)	0.39 (0.20, 0.55)
Signal-to-noise ratio	14.4±2.9	13.9±2.6	14.5±2.9	0.72 (0.60, 0.81)	0.74 (0.63, 0.82)
Contrast-to-noise ratio	21.3±4.7	21.8±5.0	21.6±4.8	0.63 (0.48, 0.74)	0.69 (0.56, 0.78)
Figure of merit	376.5±224.7	401.5±255.6	396.0±267.6	0.77 (0.67, 0.84)	0.80 (0.72, 0.87)
Descending aorta					
Mean attenuation	244.1±37.7	244.9±38.1	244.8±37.5	0.99 (0.98, 0.99)	0.99 (0.98, 0.99)
Standard deviation	18.8±2.7	18.5±3.0	18.6±2.7	0.51 (0.34, 0.65)	0.53 (0.36, 0.66)
Signal-to-noise ratio	13.2±2.5	13.5±2.8	13.4±2.6	0.70 (0.58, 0.79)	0.76 (0.66, 0.83)
Contrast-to-noise ratio	20.6±4.6	21.3±4.9	21.1±4.8	0.66 (0.52, 0.76)	0.69 (0.57, 0.79)
Figure of merit	354.5±213.3	383.7±249.4	378.0±259.1	0.78 (0.69, 0.85)	0.81 (0.72, 0.87)
Pulmonary trunk					
Mean attenuation	250.7±53.2	252.0±53.8	252.0±52.3	0.98 (0.97, 0.99)	0.98 (0.97, 0.99)
Standard deviation	17.9±2.3	18.4±3.0	17.8±2.4	0.58 (0.43, 0.70)	0.61 (0.46, 0.72)
Signal-to-noise ratio	14.1±3.1	14.0±3.4	14.3±3.2	0.82 (0.73, 0.87)	0.86 (0.80, 0.91)
Contrast-to-noise ratio	21.1±5.5	21.7±5.4	21.6±5.5	0.71 (0.59, 0.80)	0.74 (0.63, 0.82)
Figure of merit	378.1±260.1	401.0±270.4	399.9±294.7	0.81 (0.73, 0.87)	0.81 (0.73, 0.87)
Erector spinae					
Mean attenuation	52.4±11.3	57.6±6.9	51.0±11.7	0.36 (0.14, 0.54)	0.71 (0.59, 0.80)
Standard deviation	23.2±3.2	20.7±3.5	23.8±4.0	0.22 (0.01, 0.41)	0.37 (0.18, 0.53)
Signal-to-noise ratio	2.3±0.7	2.9±0.6	2.2±0.7	0.32 (0.02, 0.55)	0.60 (0.45, 0.72)
Contrast-to-noise ratio	9.2±1.9	9.9±2.0	9.4±2.0	0.47 (0.29, 0.62)	0.57 (0.41, 0.69)
Figure of merit	67.2±32.6	77.3±37.5	71.0±42.2	0.58 (0.41, 0.70)	0.65 (0.52, 0.76)
Axillary fat					

Mean attenuation	-102.5±10.5	-104.8±9.1	-103.9±9.1	0.65 (0.51, 0.76)	0.70 (0.58, 0.79)
Standard deviation	17.4±3.1	17.0±3.2	17.1±3.1	0.53 (0.36, 0.66)	0.54 (0.38, 0.67)
Signal-to-noise ratio	-6.1±1.4	-6.4±1.4	-6.3±1.4	0.55 (0.39, 0.67)	0.62 (0.47, 0.73)

Note: ICC model of inter-rater: two-way random-type absolute agreement; ICC model of intra-rater: two-way mixed effects model, absolute agreement. ICC, intraclass correlation coefficient; CI, confidence interval.

Table S7. Reference values for the interpretation of the intra-class correlation coefficient

Correlation coefficient	Strength of reliability
<0.50	Poor reliability
0.50–0.75	Moderate reliability
0.75–0.90	Good reliability
>0.90	Excellent reliability

Table S8. Comparing Radiation Between Groups

	Group 1 Standard 320-120kVP (n=92)	Group 2 320-100kVP (n=92)	Group 3 270-100kVP (n=93)	Group 4 240-100kVP (n=93)	p value
CT dose index [mGy]	2.5(2.2-3.0)	2.1(1.7-2.5)	2.1(1.7-2.7)	2.2(1.8-2.7)	<0.001
Scan length [cm]	41.5(39.4-45.2)	42.7(40.0-45.6)	43.0(40.7-46.2)	43.7(41.3-47.1)	0.043
Dose length product value [mGycm]	109.7(88.0-127.4)	92.4(72.4-106.2)	89.5(72.9-112.3)	92.8(73.1-119.0)	<0.001
Effective dose [mSv]	1.5(1.2-1.8)	1.3(1.0-1.5)	1.3(1.0-1.6)	1.3(1.0-1.7)	<0.001

Data are presented as the median (IQR). P-values were calculated using the Kruskal-Wallis test. The effective dose was calculated using a conversion factor of 0.014.
